# Acid-modified biochar combined with reduced nitrogen fertilizer improves soil organic carbon and cotton yield in saline-alkali soil

**DOI:** 10.3389/fpls.2026.1843141

**Published:** 2026-07-14

**Authors:** Shining Liu, Yongjie Wei, Xiaofeng Wang, Jing Yang, Jiao Lin, Wenqing Zhao, Wei Hu, Mingwei Du, Zhiguo Zhou, Sumei Wan

**Affiliations:** 1 College of Agriculture, Tarim University, Alar, China; 2 Key Laboratory of Genetic Improvement and Efficient Production for Specialty Crops in Arid Southern Xinjiang of Xinjiang Corps, Tarim University, Alar, China; 3 College of Agriculture, Nanjing Agricultural University, Nanjing, China; 4 College of Agronomy and Biotechnology, China Agricultural University, Beijing, China

**Keywords:** acid-modified biochar, microbial carbon use efficiency, nitrogen fertilization, soil organic carbon, soil quality

## Abstract

**Background:**

Saline-alkali soils commonly face the dual challenges of low stock and activity in soil organic carbon (SOC), and long-term excessive nitrogen (N) fertilizer application further exacerbates soil quality degradation. Acid-modified biochar offers a promising approach for improving the fertility of saline-alkali soils, yet the effects of its combined application with N fertilizer on SOC fractions and soil quality remain insufficiently studies.

**Methods:**

Therefore, we conducted a two-year field experiment to comprehensively assess the effects of acid-modified biochar combined with N fertilizer application on soil quality index (SQI), microbial nutrient limitation and microbial carbon use efficiency (CUE), SOC fractions and yield. A randomized complete block experiment with five treatments was established: no chemical N fertilizer and no acid-modified biochar (CK), 300 kg N ha^−1^ (N1), 450 kg N ha^−1^ (Recommended N application rate, N2), 300 kg N ha^−1^ + acid-modified biochar (BN1), and 450 kg N ha^−1^ + acid-modified biochar (BN2).

**Results:**

The BN1 and BN2 treatments effectively lowered soil pH by 3.7%–5.6% and increased the contents of available phosphorus (Av-P), available potassium (Av-K), nitrate nitrogen (NO_3_^−^-N), ammonium nitrogen (NH_4_^+^-N), and microbial biomass nitrogen (MBN). These improvements collectively resulted in a SQI that was 4.0–5.1 times higher than that in the N1 and N2 treatments. Furthermore, the BN1 and BN2 treatments reversed the suppression of soil enzyme activities induced by sole N application, enhanced microbial CUE by 71.7%–105.2%, and mitigated microbial C limitation. Compared with N2, BN1 and BN2 treatments increased SOC by 35.5%–51.2%, easily oxidizable carbon (EOC) by 1.2–2.7 folds, and microbial biomass carbon (MBC) by 34.4%–46.1%. The cotton yield under BN treatments exceeded that of N2 by 10.1%–15.5% in two years. No significant differences were observed between BN1 and BN2 in terms of SQI, microbial CUE, and yield. Path analysis confirmed that fertilization-induced alterations in soil properties indirectly promoted soil enzyme activities and microbial CUE, which not only facilitated SOC accumulation but also directly boosted yield.

**Conclusion:**

Overall, 20% reduction of conventional N fertilizer combined with acid-modified biochar was more effective at improving soil quality and reducing microbial C limitation, enhance microbial CUE, and foster the accumulation of SOC, providing guidance for sustainable development in saline-alkali soil.

## 1 Introduction

Soil organic carbon (SOC) is both a cornerstone of soil health and a key component of the global carbon cycle, serving as a critical link between climate change mitigation and agricultural sustainability (Garsia et al., 2023). However, the soil organic C density of farmland soils in China (32.5 t ha^-1^) is only 64% of the global average (50.4 t ha^-1^), with even lower densities in saline-alkali soil regions, dropping to 18.7 t ha^-1^ (IPCC, 2007; Lal, 2004). Furthermore, saline-alkali soils are characterized by high salinization, strong alkalinity and low microbial activity, which leads to slow organic C transformation and a low proportion of labile fractions (Sahab et al., 2021). Nitrogen (N) is an indispensable nutrient for crop growth and development. The application of chemical N fertilizer has long served as a key strategy to increase crop yield in salinized soils and to mitigate the imbalance in SOC decomposition (Brown et al., 2014; Yuan et al., 2021). Nevertheless, long-term excessive application of chemical N fertilizer not only results in low N use efficiency and deterioration of soil physical and chemical properties but also often induces soil nutrient imbalance, microbial resource limitation, and ultimately weakens SOC sequestration capacity in agricultural ecosystems (Zheng et al., 2019). Therefore, exploring management strategies that synchronously sustain crop yield, promote soil organic carbon accumulation and improve saline-alkali soil quality is vital to the sustainable development of agriculture in saline-alkali regions.

As a C-rich product derived from biomass pyrolysis, biochar exhibits substantial potential in improving soil quality, increasing SOC content, reducing greenhouse gas emissions, and boosting crop yields (Schmidt et al., 2021; Liu et al., 2023; Yuan et al., 2025). However, biochar has inherent alkalinity and high salt ion content. When applied to salinized soils, it tends to further increase soil pH and aggravate salt stress, which in turn inhibits SOC accumulation and microbial transformation efficiency, restricting its application effect (Dai et al., 2021; Joseph et al., 2021; Liao et al., 2022). Acid modification performs functional modification on the biochar surface through acid solutions, which can increase the number of oxygen-containing functional groups such as carboxyl and hydroxyl groups (Houben and Sonnet, 2015), reduce the pH of biochar itself, thereby alleviating the alkaline stress when applied to saline soils. For example, He et al. (2023) demonstrated that the chemical and biological characteristics of saline-alkali soil are improved by the combined application of acidic biochar, which greatly increases rhizosphere soil nutrient availability and fertilizer use efficiency. Zhou et al. (2021) showed that acidic corn stalk biochar exerted positive effects on the salt distributions in soil and the sorghum yields in saline-alkali soils on the Songnen Plain of China. This may creates a more favorable soil environment for SOC stabilization and microbial-mediated carbon conversion processes, ultimately enhancing SOC sequestration (Ma et al., 2026). Most existing studies focus on alkaline biochar (Zhao et al., 2026; Liao et al., 2019), and few studies have examined SOC responses to sole acid-modified biochar or its combination with N fertilization.

Microorganisms play a central role in regulating soil C cycle (Tayyab et al.,2021). Microbial carbon use efficiency (CUE), which measures the proportion of exogenous organic matter and SOC assimilated into microbial biomass (Tao et al., 2023), is a key parameter determining carbon flow direction and sequestration potential. Low CUE increases respiratory losses and hinders SOC formation, whereas high CUE promotes carbon sequestration in the form of microbial necromass (Domeignoz-Horta et al., 2020; Yang et al., 2025). However, the effects of acid-modified biochar on CUE remain poorly understood. A complex interplay of mechanisms underpins CUE regulation following biochar addition, covering soil property modification (Cheekilote et al., 2025), microbial community shifts (Ramesh et al., 2019) and nutrient variation (Schleuss et al., 2019). Such biochar additions improve soil structure and resource availability through enhancing aggregate formation and nutrient supply. However, the formation of soil aggregates (physical protection) may limit microbial access to substrates and constrain C assimilation. Soil extracellular enzyme stoichiometry acts as a “biological bridge” connecting microbial metabolic demand with soil C cycling processes (Lasota et al., 2022). By reflecting the nutrient limitation status of microorganisms, it regulates the decomposition and transformation of SOC, making it an indispensable core indicator for understanding SOC dynamics. In addition, when the stoichiometric ratio of soil extracellular enzymes does not align with microbial demands, excess C availability exacerbates N and phosphorus (P) limitations, thereby stimulating microbial decomposition of SOC to acquire nutrients, which ultimately hinders the formation of SOC (Mao et al., 2024; Parvez and Ahammed, 2024). Variations in soil extracellular enzyme stoichiometry can impact microbial metabolism, enzymatic activity, and CUE. Wang et al. (2026) showed that manure promoted mineral-associated organic carbon and improved CUE through enhanced biomass turnover and carbon allocation. However, it is not clear how acid-modified biochar addition combined with N fertilizer influence soil extracellular enzyme stoichiometry, CUE, and SOC fractions in saline-alkali soil.

Therefore, the objectives were to: (1) investigate the effects of acid-modified biochar and N fertilization on soil enzyme stoichiometry, microbial CUE, and SOC; (2) to elucidate the relationships among SQI, soil enzyme stoichiometry, microbial CUE and SOC under acid-modified biochar and N fertilization. We hypothesized that acid-modified biochar combined with N fertilization could improve soil quality, alleviate microbial nutrient limitation, optimize soil enzyme stoichiometry, improve microbial CUE, and thereby enhance soil organic carbon accumulation and cotton yield in saline-alkali soil. This study provides field-based evidence for acid-modified biochar application and N fertilization management in saline–alkali farmlands of arid and semi-arid regions.

## 2 Materials and methods

### 2.1 Study location

The field experiment was conducted from 2024 to 2025 in Southern Xinjiang Industry-Education Integration Modern Agriculture Training Base of Tarim University (44°32′N, 81°18′E), Alar, China. This area features a warm-temperate continental arid desert climate, boasting an average annual temperature of 10.7°C and receiving only 40.1–82.5 mm of rainfall. The soil was classified as sandy loam. Soil physicochemical properties of 0–20 cm topsoil before cotton sowing are given in **Table 1**.

**Table 1:** Soil physical and chemical properties before cotton sowing.

Properties	Quantity	Units
Bulk density	1.54	g cm^−3^
pH (H_2_O)	7.9	–
TN	0.82	g kg^−1^
TP	113.1	mg kg^−1^
Av-N	101	mg kg^−1^
Av-P	12.3	mg kg^−1^
Av-K	135	mg kg^−1^
Organic matter	16.95	m kg^−1^

TN, total nitrogen; TP, total phosphorus; Av-P, available phosphorus; Av-N, available nitrogen; Av-K, available potassium.

### 2.2 Experimental design

The experiment was conducted in a randomized block design with five treatments: no chemical N fertilizer and no acid-modified biochar (CK), 300 kg N ha^−1^ (N1), 450 kg N ha^−1^ (Recommended N application rate, N2), 300 kg N ha^−1^ + acid-modified biochar (BN1), and 450 kg N ha^−1^ + acid-modified biochar (BN2). Each treatment was replicated four times, resulting in 20 plots and measuring 92 m^2^ (4.6 m × 20 m). Biochar applied at 15 t ha^−1^ was incorporated into the same position using a rotary tiller at a 20–30 cm before cotton sowing in April 2023. Based on our preliminary trials (unpublished) and previous reports in arid and semi-arid saline-alkali soils (Ren et al., 2025), a biochar rate of 15 t ha^-^¹ was found to be optimal and was thus adopted as the sole rate in this experiment to examine its combined effects with different N application rates. The biochar were purchased from Henan Dakang Water Purification Materials Co., Ltd. The original biochar was separately immersed in 0.3 mol·L^−1^ citric acid solution with the solid-to-liquid ratio controlled at 1:50 (g:mL). The mixture was magnetically stirred at 800 r min^−1^ for 4 hours at room temperature. After stirring, the samples were dried in an oven at 65°C for 24 h, and then heat-treated at 120°C for 90 min to trigger esterification reaction. After cooling, the modified biochar was repeatedly washed with deionized water until the pH value of the filtrate remained stable. Finally, the samples were dried at 65°C to obtain acid-modified biochar (Yan et al., 2016).

Cotton (cultivar Tahe No.2) was sown on 15 April 2024 and 20 April 2025, and harvested in the early part of October each year. Cotton was sown using a wide-narrow row arrangement (66 cm + 10 cm) at a planting density of 220, 000 plants ha^−1^, and the spacing between cotton plants was 11.5 cm. Urea (46% N), diammonium phosphate (46% P_2_O_5_, 150 kg P_2_O_5_ ha^–1^) and potassium chloride (60% K_2_O, 75 kg ha^−1^) were applied distributed proportionally in accordance with the cotton key growth stages as water. Six irrigation events were conducted per year for each block, and each event had an application rate of 635 m^3^ ha^−1^.

### 2.3 Soil properties

For soil sampling, six random subsamples were collected from each individual plot and thoroughly homogenized into one composite sample to reduce spatial soil heterogeneity. In this study, the six subsamples within a single plot were defined as technical sampling replicates, whereas the four independent plots for each treatment were regarded as biological replicates. All soil physicochemical and microbial indicators, including SOC, DOC, MBC, MBN, enzyme activities, and CUE, were determined using these four composite samples. Each soil sample was divided into two portions. One portion was air-dried and sieved through a 2 mm mesh to determine soil total carbon (TC), total nitrogen (TN), total phosphorus (TP), available phosphorus (Av-P), available potassium (Av-K), and soil organic carbon fraction. The other fresh soil was cleared of stones, plant residues and debris, then sieved for the measurement of ammonium nitrogen (NO_3_^−^-N), nitrate nitrogen (NH_4_^+^-N), microbial biomass carbon (MBC), microbial biomass nitrogen (MBN), soil enzyme activities and carbon use efficiency (CUE).

Soil TC and TN contents were determined using a soil carbon-nitrogen elemental analyzer (Elementar Vario MAX CN, Germany). Soil TP was determined by sodium hydroxide fusion-molybdenum-antimony anti-spectrophotometric method. Soil NO_3_^−^-N and NH_4_^+^-N were extracted with 0.5 M K_2_SO_4_ and determined by the colorimetric assay.

Soil organic carbon (SOC) was measured using concentrated sulfuric acid-potassium dichromate. Easily oxidizable carbon (EOC) was extracted with 333 mmol L^−1^ potassium permanganate (Blair et al., 1995) and measured by an ultraviolet-visible spectrophotometer (Shimadzu UV-1800, Japan). Dissolved organic carbon (DOC) was extracted from soil with distilled water and analyzed using a total organic carbon analyzer (Shimadzu TOC-VCPH, Japan) (Liu et al., 2022). Microbial biomass carbon (MBC) and microbial biomass nitrogen (MBN) were determined via the chloroform fumigation-K_2_SO_4_ extraction method (Blair et al., 1995). The extract was filtered through a 0.45 μm filter membrane before measurement with a total organic carbon analyzer (Shimadzu TOC-VCPH, Japan) (Lu, 2000).

### 2.4 Soil extracellular enzyme activity

Soil enzyme activities, including β-1, 4-glucosidase (BG), cellobiohydrolase (CBH), β-1, 4-N-acetylglucosaminidase (NAG), leucine aminopeptidase (LAP), and alkaline phosphatase (AP), were quantified using a standardized fluorescence method. Briefly, 2 g fresh soil was homogenized in 125 mL of distilled water and shaken continuously at 180 rpm for 2 h at 25°C to obtain a uniform soil suspension.The sample suspension, a substrate solution (BG, 4-Methylumbelliferyl-β-Dglucoside; CBH, 4-Methylumbelliferyl-β-Dcellobioside; NAG, 4-Methylumbelliferyl-N-acetyl-β-Dglucosaminide; LAP, L-Leucine-7-amido-4-methylcoumarin hydrochloride; AP, 4-Methylumbelliferyl phosphate), and a buffer were injected into a 96-well enzyme standard plate in a specific sequence using a multichannel pipette. All samples were incubated in darkness at 25°C for 4 h to ensure sufficient enzymatic reaction. After incubation, 50 μL of 0.5 mol L NaOH solution was added to each well to terminate the enzymatic reaction. The reacted solution was then transferred to new labeled microplates for fluorescence detection. Enzyme activities were expressed as the amount of substrate converted per gram of sample per hour (μmol g^-^¹ h^-^¹) (Marx et al., 2001).

Soil enzyme stoichiometry vectors were used to determine the potential and relative extent of soil microbial C, N, and P limitation. Vector length reflects relative C limitation with respect to nutrients (N/P), while vector angle indicates relative N/P limitation (Li et al., 2022).

(1) $$\text{Vector length}={\left({\text{x}}^{2}+{\text{y}}^{2}\right)}^{\frac{1}{2}}$$

(2) $$\text{Vector Angle}=\text{atan}2(\text{x},\text{y})\times \left(\frac{180}{\pi }\right)$$

Where, x and y denote the relative activities of C-acquiring enzymes and N/P-acquiring enzymes, respectively. These were calculated as ln(BG:(BG + NAG + LAP)) and ln(BG:(BG + AP)) based on ln-converted net activities. Microbial C limitation intensified with increasing vector length, while vectors >45° or<45° indicated microbial nitrogen or phosphorus limitation, respectively.

### 2.5 Soil quality index and microbial carbon use efficiency

For all experimental treatments, the soil quality index (SQI) was determined by normalizing soil properties to non-weighted scores (ranging from 0 to 1) using the following equation (Yan et al., 2024):

(3) $${\text{SL}}_{\text{i}}=({\text{X}}_{\text{i}}-{\text{L}}_{\text{i}})/({\text{H}}_{\text{i}}-{\text{L}}_{\text{i}})$$

Where, SLi represents the linear transformation value of soil properties. Xi represents the observed value of soil properties, with Hi and Li being the corresponding maximum and minimum values. Soil properties (pH, SOC, TN, TP, Av-P, Av-K, DOC, NH_4_-N, NO_3_-N, and EOC), enzyme activities (BG, NAG, AP, LAP), and microbial biomass (MBC and MBN) were used to calculate the SQI. The SQI-area method was ued to determine the SQI, with the calculation relying on the triangular area in the radar map derived from processed soil properties (Kuzyakov et al., 2020).

(4) $$\text{SQI }=\;0.5\;\times \sum_{\text{i}=1}^{\text{n}}{\text{SL}}_{\text{i}}^{2}\;\times \text{ sin}\frac{2\text{π}}{\text{n}}$$

Here, n denotes the quantity of soil properties incorporated into the SQI calculation.

The microbial carbon use efficiency (CUE) was determined using the ^18^O–H_2_O method (Schroeder et al., 2024). 10 g fresh soil sample from each treatment were pre-incubated for 24 hours at 15°C under a water holding capacity (WHC) of 60%, and divided the soil sample into two sub-sample. One sub-sample was transferred into a 2 mL screw-cap vial, and 50–60 μL of ^18^O-labeled water (97.0 atom% ^18^O) was added to achieve a final soil water ^18^O enrichment of 20 atom%; other sub-sample was mixed with an equal volume of Milli-Q water and served as an unlabeled control. Subsequently, the screw-cap vial was transferred into a 20 mL headspace vial, which was sealed for 5 min, flushed with CO_2_-free air, and incubated at 15°C for 24 h. Thereafter, the CO_2_ concentration (soil respiration, Rs, in ng C g^-1^ h^-1^) in the 20 mL headspace vial was measured using a gas chromatograph (GC-7890B, Agilent, USA). The soil sample was then immediately lyophilized, and total DNA was extracted using a commercial DNA extraction kit (PowerSoil, MoBio). The DNA concentration in an aliquot of the purified extract was quantified via the PicoGreen fluorescence assay (PicoGreen, ThermoFisher) with a microplate spectrophotometer (Infinite M200, Tecan, Austria). Subsequently, the remaining DNA extract was dried at 45°C for 5 h in a silver capsule to remove residual moisture, and the ^18^O and total oxygen content was analyzed using an isotope ratio mass spectrometer coupled with a high-temperature conversion elemental analyzer (IRMS-TC/EA, Thermo Scientific, TX, USA).

Microbial growth rate (Mg) was determined by calculating DNA production during soil incubation. Based on the differences in ^18^O-labeled DNA levels between unlabeled and ^18^O-labeled soil samples, DNA production (DNAp, μg) was calculated according to Canarini et al. (2020) and Qu et al. (2020):

(5) $${\text{DNA}}_{\text{P}}={\text{O}}_{\text{t}}\times \frac{\text{at}{\%}_{\text{e}}}{100}\times \frac{100}{\text{at}{\%}_{\text{f}}}\times \frac{100}{31.21}$$

Here, Ot represents the total O content (μg) in the dried DNA extract, at%e represents the difference in the at% ^18^O levels between the nonlabeled and labeled samples, at%f is the ^18^O atom % of soil water before incubation (20% in the present study), and 31.21 represents the mean O level in the DNA (C_39_H_44_O_24_N_15_P_4_). The O in freshly extracted DNA was assumed to originate solely from water.Additionally, new ^18^O-labeled microbial cell mortality was considered negligible given the short incubation period (Qu et al., 2020; Schimel et al., 2022).

(6) $$M_{g}=\frac{f_{\mathrm{DNA-MBC}}\times \mathrm{DNAp}\times 1000}{\mathrm{DW}\times t}$$

Where, f_DNA-MBC_ is the ratio of soil MBC: DNA content, DW (g) is the dry weight of soil.

The microbial respiration rate (Rs, ng C g^−1^h^−1^) was measured as follows (Schimel et al., 2022);

(7) $${\text{R}}_{\text{S}}=\frac{\text{Rc}}{\text{DW}\times \text{t}}\times \frac{\text{P}\times \text{N}}{\text{R}\times \text{T}}\times \text{V}\times 1000$$

Where, P is the atmospheric pressure (kPa), n is the element C molecular mass (12.01 g mol^−1^), R is the ideal gas constant (8.314 J mol^−1^ K^−1^), and T is the absolute temperature of the gas (295.15 K). V is the headspace volume of the vial (L). Rc (ppm) refers to the CO_2_ concentration produced during the 24-hour incubation period.

CUE was calculated as follows (Sinsabaugh et al., 2016):

(8) $$\text{CUE}=\frac{{\text{M}}_{\text{g}}}{{\text{M}}_{\text{g}}+{\text{R}}_{\text{s}}}$$

### 2.6 Cotton yield

Three 1 m^2^ sampling subplots were randomly chosen from the center of each plot. Cotton bolls in each treatment were then collected manually. The weight of individual bolls and the number of effective bolls per plant were carefully documented to calculate seed cotton yield. The lint yield was obtained after ginning.

### 2.7 Statistical analysis

Microsoft Excel 2019 was used for data organization, and SPSS 23.0 for statistical analysis. One-way analysis of variance (ANOVA) and the Tukey’s HSD *post-hoc* test were applied to test the significance of different index under different treatments. The stoichiometric ratio of C-to-N acquiring enzymes versus C-to-P acquiring enzymes, vector length and angle were calculated using the R package ‘Vegan’. To assess the direct and indirect impacts of N fertilizer and acid-modified biochar addition on soil organic carbon fractions, soil properties, soil enzymes, microbial carbon use efficiency (CUE) and cotton yield, partial least squares path modeling (PLS–PM) was conducted using the “plspm” package in R Studio. A total of 1000 bootstrap resampling iterations were implemented to validate the stability of the pathway coefficients in the PLS-PM model. The model goodness-of-fit (GOF) statistic, ranging from 0 to 1, was quantified as the geometric mean of the average communality and the average coefficient of determination (R^2^) for all endogenous variables. The standardized root mean square residual (SRMR) value was 0.062, the normed fit index (NFI) was 0.91, the Goodness of Fit (GOF) was 0.866. Direct effects (path coefficients) represent the direction and strength of linear relationships between variables. The figures presented in this study were produced with Origin version 2024 (Origin Lab, USA).

## 3 Results

### 3.1 Soil properties

Soil properties under different treatments were presented in **Table 2**. Compared with CK treatment, BN1 and BN2 significantly decreased pH by 3.7%–5.7% and 2.6%–6.2% respectively; however, the differences between the BN1 and BN2 treatments were not significant. Compared with N2 treatment, the BN1 treatment decreased pH by 3.7%-5.6%, and the BN2 treatment decreased pH by 4.2%-4.5% in two years, respectively. Nitrogen fertilizer input significantly improved NO_3_^−^-N and NH_4_^+^-N contents by 21.2%–79.4% and 14.7%–64.5% relative to CK treatment. Compared with N2 treatment, the BN1 treatment further increased the NO_3_^−^-N and NH_4_^+^-N by 26.0%–28.4% and 16.9%–20.4%, and the BN2 treatment increased the NO_3_^−^-N and NH_4_^+^-N by 29.2%–31.4% and 16.5%–20.9% in two years, respectively. Compared with CK treatment, nitrogen fertilizer input had no effect on the Av-P and Av-K contents, but BN1 and BN2 treatments significantly increased the Av-P and Av-K contents. Compared with CK treatment, N fertilizer input had no effect on Av-P and Av-K contents, whereas acid-modified biochar combined with N fertilizer significantly increased them. Compared with N2 treatment, the BN1 treatment increased the Av-P and Av-K contents by 41.5%–56.6% and 15.6%–20.0%, and the BN2 treatment increased the Av-P and Av-K contents by 40.4%–56.5% and 16.6%–19.4%, respectively. Acid-modified biochar combined with N fertilizer application had no significant effect on soil TN and TP. Compared with the CK treatment, N application increased MBN content by 9.5%–34.6%; however, the differences between the N1 and N2 treatments were not significant. Compared with N2 treatment, the BN1 and BN2 treatments increased the MBN content by 46.5%–69.9% and 45.8%–67.8%, respectively.

**Table 2:** Soil physicochemical properties under different fertilization treatments.

Years	Treatments	pH	TN	NO_3_^−^-N	NH_4_^+^-N	TP	Av-P	Av-K	MBN
			(g kg-1)	(mg kg-1)	(mg kg-1)	(mg kg-1)	(mg kg-1)	(mg kg-1)	(mg kg^-1^)
2024	CK	7.72 ± 0.14ab	0.82 ± 0.03a	23.86 ± 1.51d	77.15 ± 1.83d	112.9 ± 0.05a	12.14 ± 0.76b	131.59 ± 8.06b	61.81 ± 0.09c
	N1	7.82 ± 0.07a	0.85 ± 0.01a	28.91 ± 1.02c	88.52 ± 0.49c	112.8 ± 0.05a	12.96 ± 0.66b	134.34 ± 3.09b	67.74 ± 0.84b
	N2	7.87 ± 0.06a	0.86 ± 0.01a	33.89 ± 1.38b	96.15 ± 1.72b	112.9 ± 0.04a	13.02 ± 0.89b	133.80 ± 9.46b	69.10 ± 1.76b
	BN1	7.43 ± 0.19c	0.86 ± 0.11a	43.53 ± 2.40a	112.38 ± 1.68a	113.1 ± 0.08a	20.39 ± 0.62a	154.74 ± 5.24a	117.42 ± 2.17a
	BN2	7.52 ± 0.02bc	0.93 ± 0.16a	44.54 ± 1.18a	111.99 ± 1.36a	113.2 ± 0.03a	20.38 ± 0.45a	155.98 ± 7.83a	119.75 ± 2.41a
2025	CK	7.83 ± 0.05a	0.83 ± 0.05b	19.95 ± 0.14d	63.89 ± 1.29d	112.7 ± 0.11a	14.63 ± 1.13b	131.59 ± 8.53b	57.32 ± 0.85c
	N1	7.85 ± 0.03a	0.84 ± 0.01b	31.07 ± 0.51c	93.65 ± 2.09c	112.7 ± 0.07a	15.55 ± 1.08b	134.34 ± 3.46b	71.87 ± 1.32b
	N2	7.67 ± 0.17a	0.82 ± 0.01b	35.80 ± 1.32b	105.10 ± 0.82b	112.9 ± 0.02a	16.55 ± 0.93b	133.80 ± 1.06b	77.15 ± 2.31b
	BN1	7.39 ± 0.15b	0.88 ± 0.03b	45.10 ± 1.44a	126.17 ± 0.95a	113.5 ± 0.03a	23.42 ± 1.22a	154.74 ± 5.17a	113.03 ± 2.91a
	BN2	7.34 ± 0.11b	0.95 ± 0.06a	46.26 ± 1.25a	127.12 ± 1.74a	113.7 ± 0.01a	23.24 ± 0.78a	155.98 ± 6.04a	112.45 ± 0.74a

TN, total nitrogen; TP, total phosphorus; Av-P, available phosphorus; Av-K, available potassium; MBN, microbial biomass nitrogen. Different letters within the same column indicate significant difference at p< 0.05 (n = 4).

### 3.2 Soil enzyme activity

**Figure 1:**
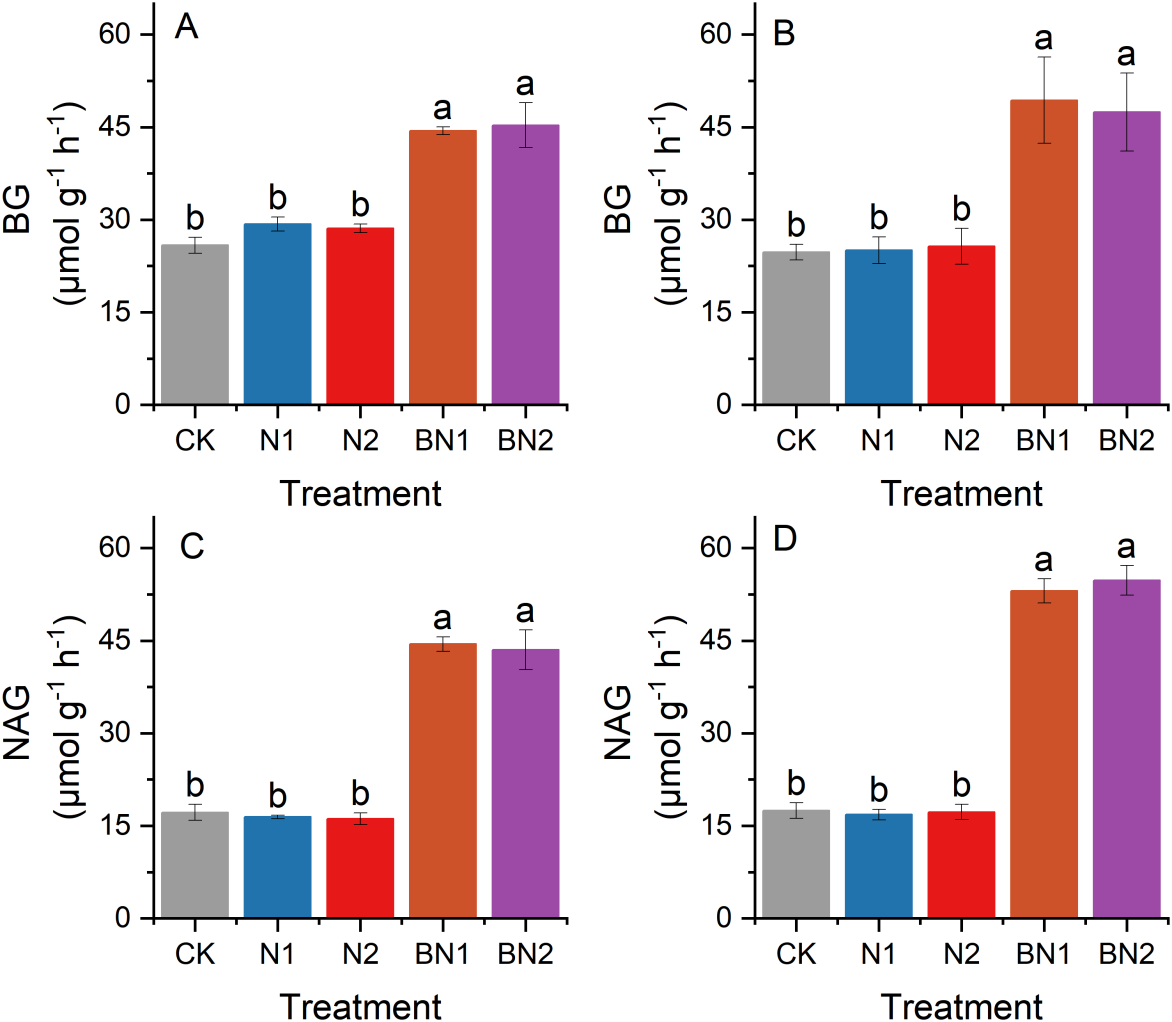
Soil β-1, 4-glucosidase [BG, **(A, B)**] andβ-N-acetylglucosaminidase [NAG, **(C, D)**] enzyme activities under different fertilization treatments in 2024 and 2025. Different lowercase letters indicate significant differences between treatments (p<0.05). Each bar represents the means (n = 4) ± SD.

**Figure 2:**
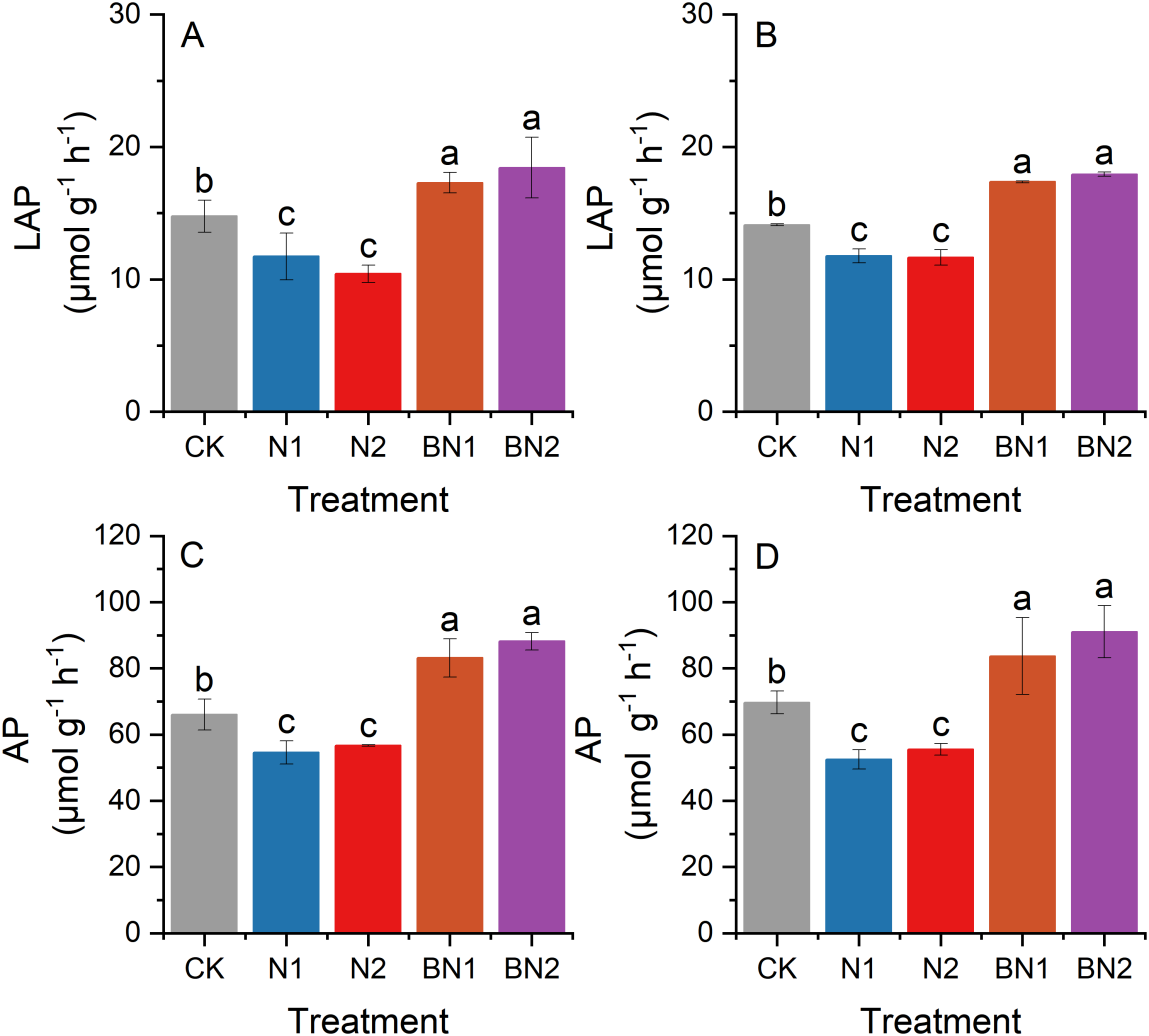
Soil leucine aminopeptidase [LAP, **(A, B)**] and alkaline phosphatase [AP, **(C, D)**] enzyme activities under different fertilization treatments in 2024 and 2025. Different lowercase letters indicate significant differences between treatments (p<0.05). Each bar represents the means (n = 4) ± SD.

Compared with CK treatment, N fertilization input treatment had no significant effect on soil β-glucosidase (BG) and N-acetyl-β-glucosaminidase (NAG) enzyme activities, whereas the combined application of acid-modified biochar and N fertilizer significantly increased these enzyme activities (**Figures 1** and **2**). The BG activity was significantly increased by 55.4%-92.2% and 58.5%-84.7% under BN1 and BN2 treatments compared to N2, respectively, in 2024 and 2025. The activity of NAG under BN1 and BN2 treatments was 1.8–2.2 times that of the N2 treatment.

Compared with CK treatment, N fertilization resulted in a significant reduction in leucine aminopeptidase (LAP) and alkaline phosphatase (AP) activities. Specifically, compared with CK, LAP activities in the N1 and N2 treatments decreased by 16.6%–20.5% and 17.4%–29.5%, respectively, while AP activities in the same treatments decreased by 17.2%–24.7% and 14.1%–20.3%, respectively. In contrast, co-applying acid-modified biochar with N fertilizer significantly increased LAP and AP activities. Compared with the N2 treatment, LAP activities under BN1 and BN2 increased by 49.0%–66.1% and 53.9%–77.1%, respectively, while AP activities increased by 46.6%–50.6% and 55.5%–63.9%, respectively. However, no significant differences were observed between the BN1 and BN2 treatments for any of the four enzyme activities.

### 3.3 Soil quality index, carbon use efficiency, and microbial resource limitations

**Figure 3:**
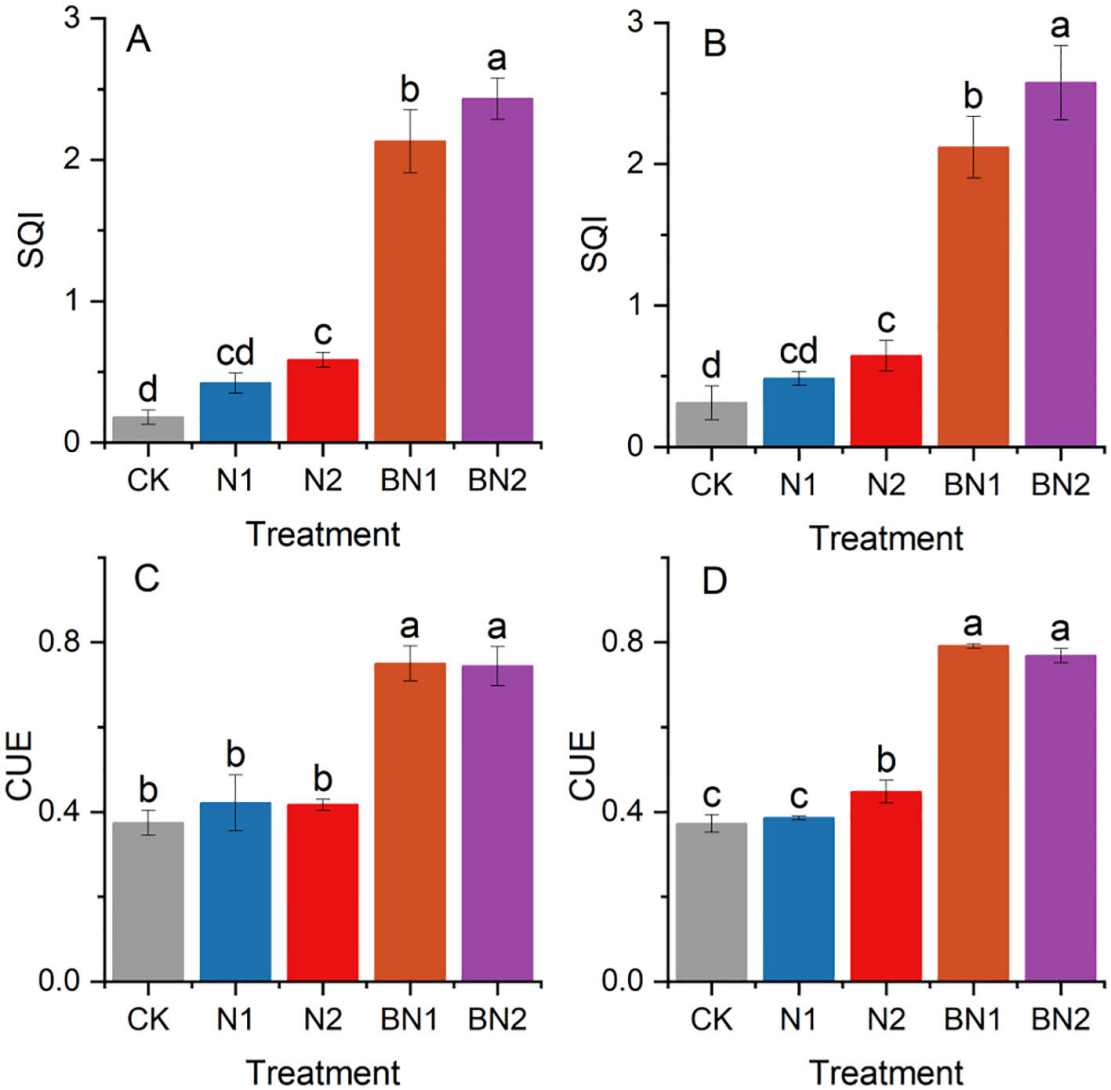
Soil quality index [SQI, **(A, B)**] and microbial carbon use efficiency [CUE, **(C, D)**] under different fertilization treatments in 2024 and 2025. Different lowercase letters indicate significant differences between treatments (p<0.05). Each bar represents the means (n = 4) ± SD.

**Figure 4:**
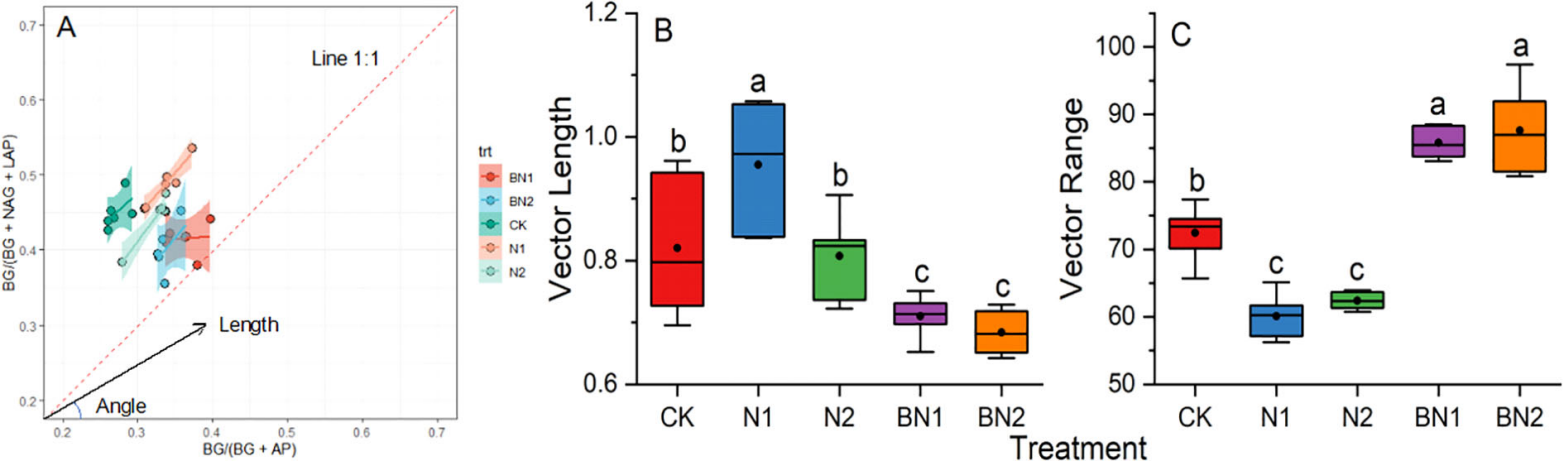
Extracellular enzyme stoichiometry of the relative proportions of C to N acquisition versus C to P acquisition **(A)**, the variation of vector length **(B)**, and angle **(C)**. Different lowercase letters indicate significant differences between treatments (p<0.05). Each bar represents the means (n = 4) ± SD.

Different fertilization treatments exerted significant effects on SQI, whereas no remarkable difference was found between N1 and N2 (**Figures 3A, B**) (Equations 3, 4). The values of BN1 and BN2 were 4.4–5.1 and 4.0–4.2 times higher than those of N1 and N2, respectively. Similarly, microbial carbon use efficiency (CUE) increased by 78.1%–105.2% in BN1 compared with N1, and rose by 71.7%–78.4% in BN2 relative to N2 (**Figures 3C, D**) (Equations 5–8). Soil enzyme stoichiometry vectors under different treatments were presented in **Figure 4A**. All data were above the 1:1 dashed line. Differences in vector length (0.684–0.955) and angle (60.113°–100.9°) were detected between treatments, with the N1 treatment had the longest vector length (microbial C limitation), while BN2 treatment showed the shortest vector length (**Figures 4B, C**) (Equations 1, 2). No significant difference was observed in vector length between the BN1 and BN2 treatments. The maximum vector angle was observed under the BN2 treatment.

### 3.4 Soil organic carbon fraction

**Figure 5:**
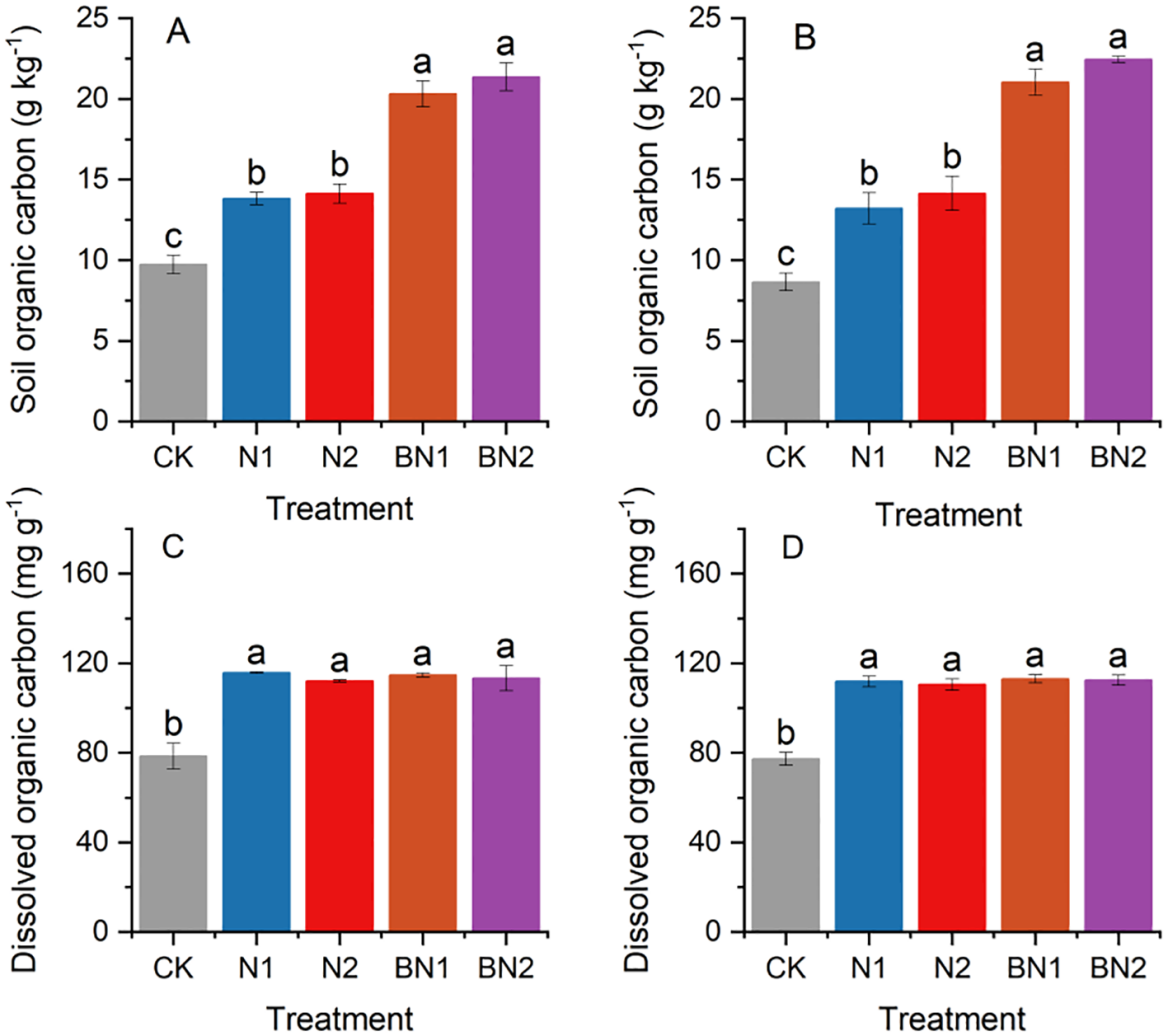
Soil organic carbon **(A, B)** and dissolved organic carbon **(C, D)** under different fertilization treatments in 2024 and 2025. Different lowercase letters indicate significant differences between treatments (p<0.05). Each bar represents the means (n = 4) ± SD.

**Figure 6:**
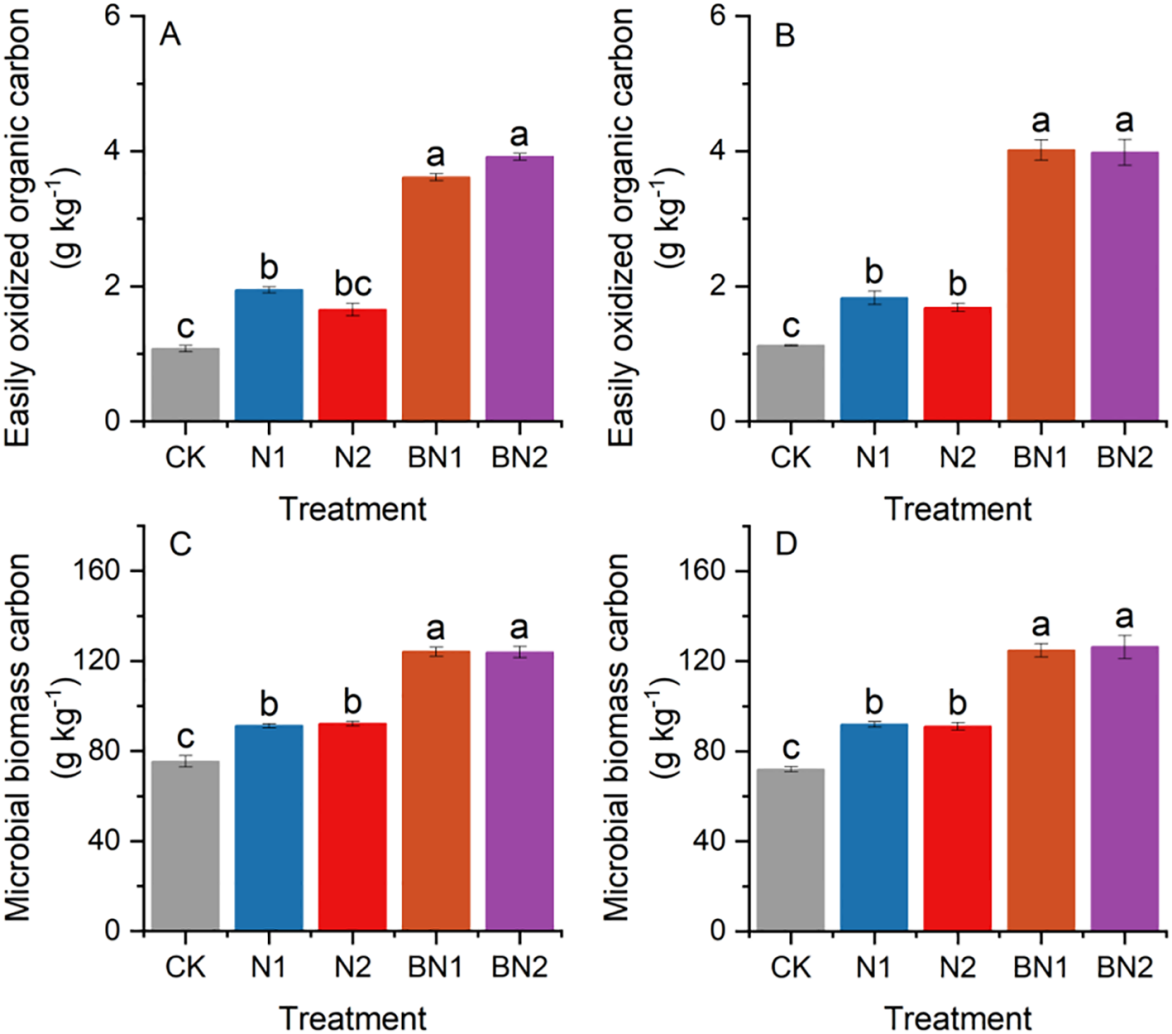
Soil easily oxidized organic carbon **(A, B)** and microbial biomass carbon **(C, D)** under different fertilization treatments in 2024 and 2025. Different lowercase letters indicate significant differences between treatments (p<0.05). Each bar represents the means (n = 4) ± SD.

Nitrogen fertilizer application significantly increased the soil organic carbon (SOC), easily oxidized organic carbon (EOC), and microbial biomass carbon (MBC) contents, but the differences between the N1 and N2 treatments were not significant (**Figures 5** and **6**). Compared with CK treatment, N fertilizer input increased the SOC and EOC contents by 37.2%–47.5% and 49.8%–80.5%, respectively, and increased MBC content by 20.9%–27.7%. Compared with N2 treatment, the BN1 treatment increased SOC content by 35.5%-39.0%, and the BN2 treatment increased SOC content by 36.2%-51.2% in two years, respectively. The EOC content under BN1 and BN2 treatments were 1.2–1.8 and 1.4–2.7 times that of the N2 treatment, respectively. A similar trend was also observed for MBC, with BN1 and BN2 treatments increasing by34.7%-37.0% and 34.4%-46.1% relative to N2, respectively. The DOC content under N1, N2, BN1, and BN2 treatments was 42.9%–46.2% higher than that under CK, but no significant differences were observed among the four treatments.

### 3.5 Cotton yield

**Figure 7:**
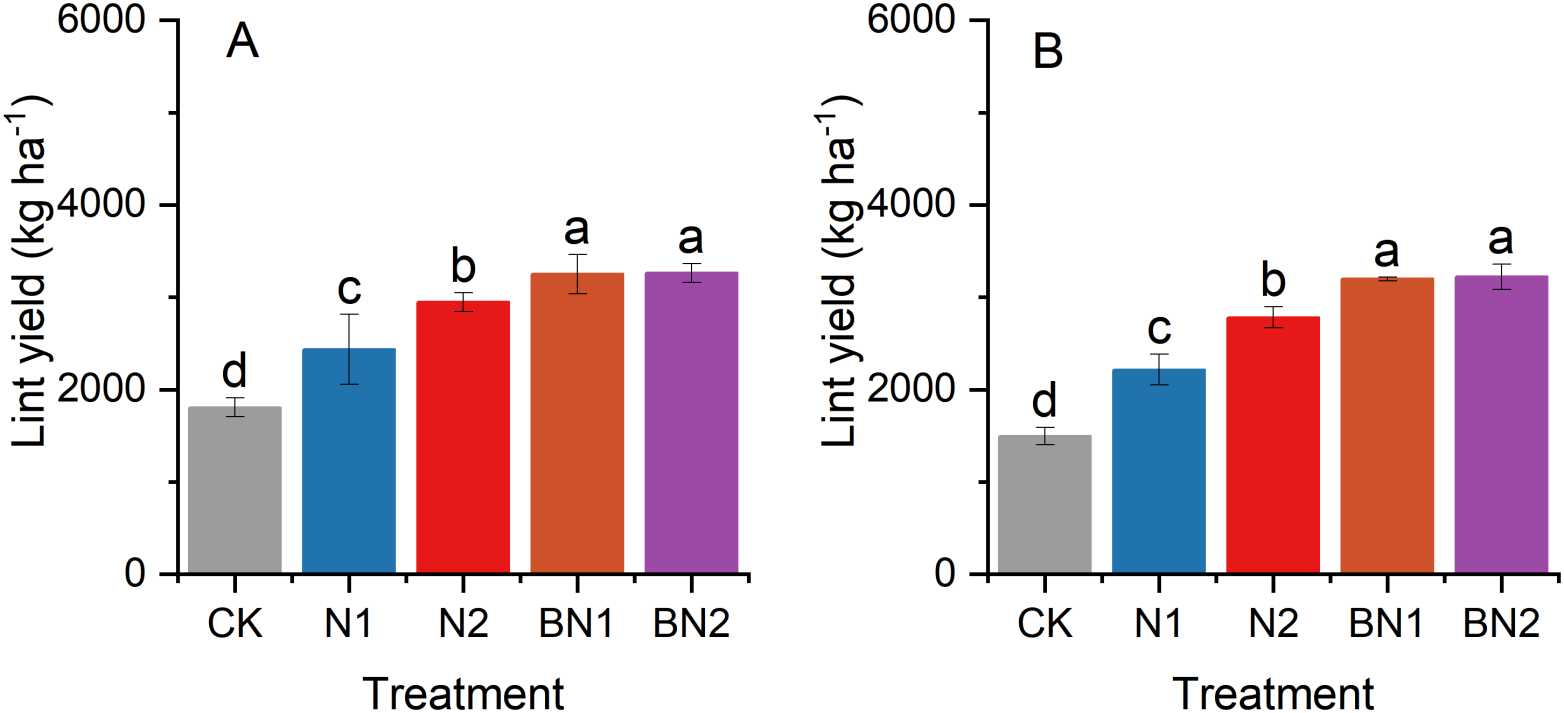
Cotton yield and quality under different fertilization treatments in 2024 **(A)** and 2025 **(B)**. Different lowercase letters indicate significant differences between treatments (p<0.05). Each bar represents the means (n = 4) ± SD.

The combined application of acid-modified biochar and N fertilizer significantly increased cotton yield; however, no significant difference in yield was observed between the BN1 and BN2 treatments (**Figure 7**). Nitrogen fertilizer treatments showed intermediate yield performance, being significantly higher than CK but substantially lower than BN1 and BN2 treatments, with N1 and N2 treatment increasing cotton yield by 35.1%–48.2% and 63.4%–86.7% relative to CK, respectively. Compared with N2 treatment, BN1 and BN2 treatments significantly increased cotton yield by 10.1%–15.1% and 10.8%–15.5% in both years, respectively.

### 3.6 Relationship among soil organic carbon fraction, soil properties, soil enzyme and microbial carbon use efficiency and yield

**Figure 8:**
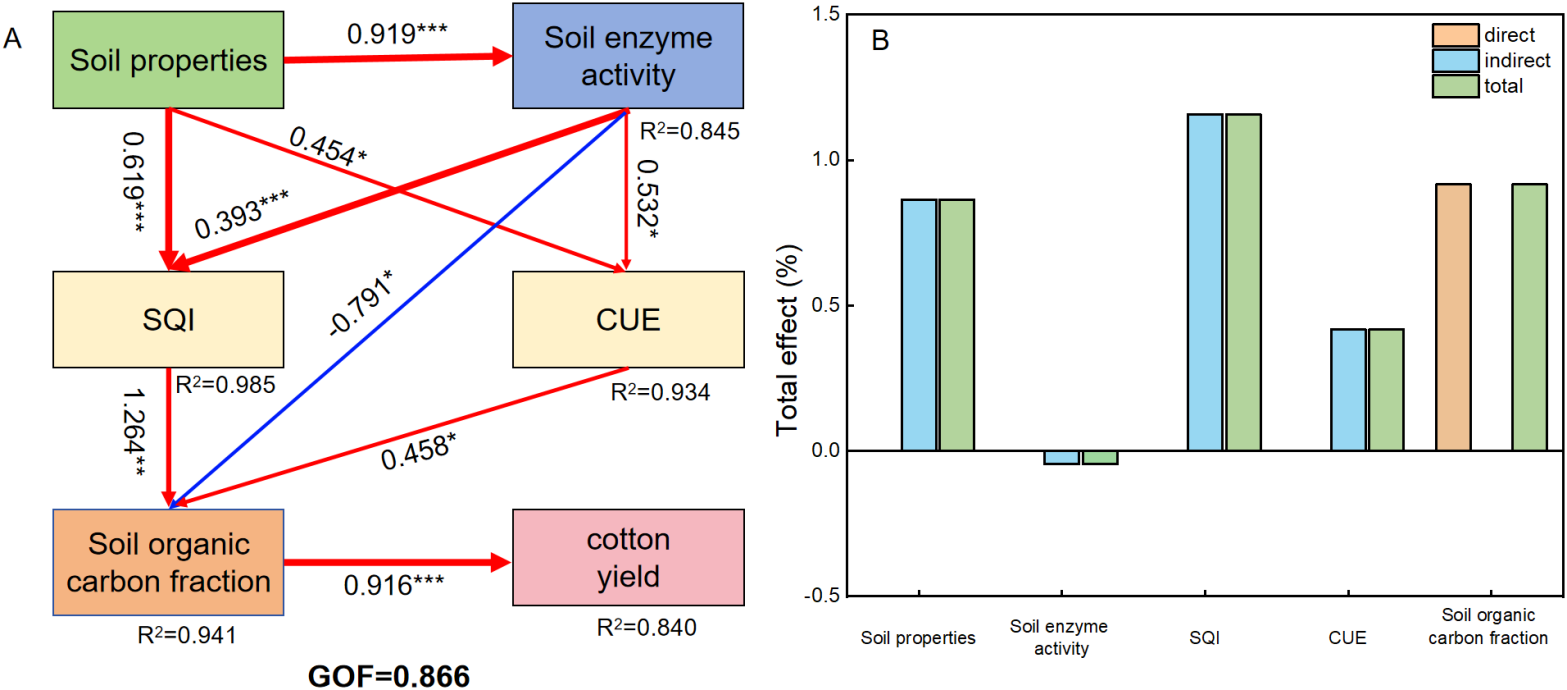
Relationships among soil organic carbon fraction, soil properties, soil enzyme and microbial carbon use efficiency (CUE) and yield estimated by partial least squares path modeling (PLS-PM) **(A)**. GOF = 0.866, SRMR = 0.062, NFI = 0.91. And the relative importance of these indexes on cotton yield **(B)**. R^2^ represents the amount of variation explained by all paths. The numbers above the arrows denote the standardized path coefficients, with solid (red) and dashed (blue) lines indicating a significant effect or no significant effect, respectively. The width of the arrows indicates the strength of the causal relationship.

A partial least squares path model was employed to establish paths for assessing the impacts of diverse variables on cotton yield (**Figure 8A**). Soil properties positively influenced by different fertilization conditions directly affect soil quality, which in turn affects soil organic C fractions and cotton yield. In addition, soil properties positively influenced soil enzyme activity, which in turn indirectly enhanced microbial CUE and consequently improved SOC fractions and cotton yield.

## 4 Discussion

The combined application of acid-modified biochar and N fertilizer effectively improved soil nutrient availability and soil quality. The positive improvement of soil quality was primarily attributed to the regulation of key soil limiting factors. On the one hand, N fertilizer provides readily available N sources, significantly increasing soil NO_3_^−^-N and NH_4_^+^-N concentrations (Pausch and Kuzyakov, 2018). The acid modification process introduces abundant carboxyl and hydroxyl acidic functional groups on the biochar surface, which effectively reduce soil pH in BN1 and BN2 treatments (Houben and Sonnet, 2015). On the other hand, acid-modified biochar directly inputs abundant labile organic compounds and releases inherent P and K nutrients into the soil. Meanwhile, its unique porous structure and rich surface functional groups can adsorb soil P and K, reduce their fixation and leaching, and ultimately improve soil nutrient availability (Lashari et al., 2013; Zhang et al., 2025; Yuan et al., 2025). In this study, the BN1 and BN2 treatments significantly increased key soil nutrient concentrations (Av-P, Av-K, NO_3_^−^-N, NH_4_^+^-N, and MBN), which directly resulted in a higher SQI (**Table 2**; **Figure 3**). This observation is in agreement with the findings reported by He et al. (2023), who reported acidic biochar and fertilizer led to synergistic improvements in soil chemical and biological properties, which significantly enhanced nutrient availability in rhizosphere soil, which directly improved the living environment for soil microbes.

The improvement of soil properties by acidic biochar also affected soil enzyme activities. In this experiment, N fertilizer only exerted no significant effects on the activities of BG and NAG, which is involved in C and N cycling. However, it significantly inhibited the activities of AP related to P cycling and LAP associated with N cycling (**Figures 1** and **2**). This might be that the simple addition of N sources exacerbated microbial P limitation or altered microbial metabolic strategies, thereby reducing enzyme investment in the acquisition of P and specific N sources. In contrast, BN1 and BN2 treatments significantly increasing the activities of BG, NAG, LAP, and AP. The possible mechanism for the increase in enzyme activity may be attributed to the increased soil C content by the acidic biochar application, which provides an organic C source for microbes (He et al., 2020). Abay et al. (2024) also confirmed that soil extracellular enzyme activity was positively correlated with soil SOC concentration following C input. However, Wang et al. (2026) concluded that the greater C inputs do not necessarily translate into higher SOC retention, as microbial carbon use efficiency is also critical for SOC sequestration. Microbial carbon use efficiency (CUE), regard as the effectiveness with which microorganisms transform substrate carbon into biomass, is essential to soil C stabilization (Geyer et al., 2016). In our study, the BN1 and BN2 treatments significantly increased microbial CUE (**Figures 3C, D**), which was consistent with the results of alleviating microbial C limitation and improving soil quaility. When microorganisms are exposed to an environment with relatively sufficient C and mitigated N and P limitations, their metabolic strategy shifts toward higher growth efficiency. Specifically, a larger fraction of assimilated carbon is allocated to microbial biomass synthesis rather than maintenance energy consumption, thereby increasing CUE (Domeignoz-Horta et al., 2020). A high CUE means that more of the assimilated C is converted into microbial biomass, while microbial necromass forms stable mineral-associated organic matter through the ‘microbial carbon pump’ pathway, becoming a major source of long-term SOC sequestration. Our findings also confirm that acid-modified biochar combined with N fertilizer had no significant effect on soil DOC, but it significantly increased EOC and MBC. Acid-modified biochar combined with N fertilizer improved microbial nutrient balance, enhanced microbial carbon use efficiency, and directed more carbon into biomass and labile forms rather than leaving it in solution as DOC.

Soil extracellular enzyme activities stoichiometric ratios are direct reflections of microbial nutrient demands (Tapia-Torres et al., 2015). Under the framework of ecological stoichiometry, microorganisms acquire C, N, and P resources by secreting different classes of extracellular enzymes (Sinsabaugh et al., 2009; Tapia-Torres et al., 2015). Therefore, the relative ratios of these enzyme activities directly indicate the types of resource limitations that microorganisms experience in a given soil environment. Numerous studies have shown that when SOC is insufficient, microorganisms invest more energy in synthesizing carbon-acquiring enzymes to accelerate organic matter decomposition and obtain C sources, which often leads to accelerated SOC mineralization and reduced sequestration (Mao et al., 2024; Mooshammer et al., 2014; Parvez and Ahammed, 2024). Conversely, when C limitation is alleviated, microorganisms can redirect more energy toward growth and reproduction processes, thereby reducing SOC decomposition rate. In this study, N1 treatment showed the longest vector length, representing severe C limitation in microorganisms; whereas acid-modified biochar combined with N fertilizer greatly reduced vector length via supplementing available C and N. Meanwhile, the vector angle under the BN2 treatment was the largest, indicating that microbial C limitation was significantly alleviated (Moorhead et al. (2016). Song et al. (2022) found that the alleviation of microbial resource limitation and the increase in CUE jointly promoted SOC accumulation, indicating that regulating microbial metabolism by improving soil nutrient availability is an effective approach to achieve C sequestration.

In this study, compared with N fertilzier only, acid-modified biochar combined with N fertilizer increased cotton yield, but no significant difference was observed between BN1 and BN2 treatments (**Figure 7**). This might be due to biochar-induced amelioration of soil properties, optimized enzyme stoichiometry, enhanced CUE, promoted SOC accumulation, and ultimately boosted yield. The results of PLS-PM further supported that acid-modified biochar combined with N fertilizer treatment improved soil properties and enzyme activities, which incredsed CUE and SOC fractions, ultimately positively increased cotton yield (**Figure 8A**). This study innovatively linked soil extracellular enzyme stoichiometry, CUE, and SOC fractions, revealing the effects of the combined application of acid-modified biochar and N fertilizer on soil enzyme stoichiometry, CUE, and SOC fractions in saline-alkali soil. These findings provide a new theoretical basis for understanding the process of soil C sequestration in saline-alkali soils under the integrated regulation of organic and inorganic amendments. There is a limitation in this experiment that the unmodified biochar treatment was not included. For this reason, the specific contribution of acid modification cannot be accurately separated from the inherent effect of biochar. Further studies will supplement raw biochar treatments under consistent nitrogen levels to distinguish the effects of biochar matrix and acid modification, so as to deeply reveal the improvement mechanism of acid-modified biochar in saline-alkali soil.

## 5 Conclusions

The combined application of acid-modified biochar and N fertilizer significantly improved soil quality and cotton yield. Compared with N fertilizer only, BN1 and BN2 treatments markedly reduced soil pH by 3.7%–5.6%, effectively neutralizing the alkalinity of saline−alkali soil. They also substantially increased soil Av−P content by 40.4%–56.6% and Av−K content by 15.6%–20.0%, while further raising the levels of NO_3_^−^-N, NH_4_^+^-N, and MBM. In addition, the BN1 and BN2 treatments effectively alleviated the suppression of soil enzyme activities caused by sole N application, leading to substantial increases in the activities of BG, NAG, LAP, and AP. Furthermore, acid-modified biochar combined with N fertilizer notably increased CUE by 71.7%–105.2% compared to the corresponding N−only treatments, thereby alleviating microbial C limitation. acid-modified biochar combined with N fertilizer had no significant effect on soil DOC, but it significantly increased EOC and MBC. Over two years, cotton yield under acid-modified biochar combined with N fertilizer increased by 10.1%–15.5% compared to the N2 treatment. However, no significant differences were observed between BN1 and BN2 treatments in terms of SQI, CUE, and cotton yield. In summary, the combined application of acid−modified biochar and N fertilizer (BN1 and BN2) promotes positive changes in soil properties. It not only directly improves soil quality and enhances the activities of C− and N−acquiring enzymes but also alleviates microbial C limitation, increases microbial CUE, and consequently promotes SOC accumulation.

## Data Availability

The original contributions presented in the study are included in the article/supplementary material. Further inquiries can be directed to the corresponding author.
